# Supplementary material for: Wide Distribution and Intraspecies Diversity in the Pathogenicity of Calonectria in Soil from Eucalyptus Plantations in Southern Guangxi of China
Source: J Fungi (Basel). 2023 Jul 29;9(8):802. doi: 10.3390/jof9080802 (PMC10455796; doi:10.3390/jof9080802)
Supplement: Supplementary file 1 [file jof-09-00802-s001.zip › jof-2453842-supplementary.pdf]

Supplementary materials

Supplementary Figure S1. Phylogenetic tree of *Calonectria* species based on maximum likelihood (ML) analyses of the *tef1* gene sequences in this study. Bootstrap support value ≥ 70% for ML and posterior probabilities values ≥ 0.95 for Bayesian inference (BI) analyses are presented above the branches as follows: ML/BI. Bootstrap values < 70% or probabilities values < 0.95 are marked with "*", and absent analysis values are marked with "-". Ex-type isolates are marked with "T". Isolates sequenced in this study are highlighted in bold and shown in color. Two isolates of *Curvicladiella cignea* (CBS 109167 and CBS 109168) were used as outgroups.

Supplementary Figure S2. Phylogenetic tree of *Calonectria* species based on maximum likelihood (ML) analyses of the *tub2* gene sequences in this study. Bootstrap support value ≥ 70% for ML and posterior probabilities values ≥ 0.95 for Bayesian inference (BI) analyses are presented above the branches as follows: ML/BI. Bootstrap values < 70% or probabilities values < 0.95 are marked with "*", and absent analysis values are marked with "-". Ex-type isolates are marked with "T". Isolates sequenced in this study are highlighted in bold and shown in color. Two isolates of *Curvicladiella cignea* (CBS 109167 and CBS 109168) were used as outgroups.

Supplementary Figure S3. Phylogenetic tree of *Calonectria* species based on maximum likelihood (ML) analyses of the *cmdA* gene sequences in this study. Bootstrap support value ≥ 70% for ML and posterior probabilities values ≥ 0.95 for Bayesian inference (BI) analyses are presented above the branches as follows: ML/BI. Bootstrap values < 70% or probabilities values < 0.95 are marked with "*", and absent analysis values are marked with "-". Ex-type isolates are marked with "T". Isolates sequenced in this study are highlighted in bold and shown in color. Two isolates of *Curvicladiella cignea* (CBS 109167 and CBS 109168) were used as outgroups.

Supplementary Figure S4. Phylogenetic tree of *Calonectria* species based on maximum likelihood (ML) analyses of the *his3* gene sequences in this study. Bootstrap support value ≥ 70% for ML and posterior probabilities values ≥ 0.95 for Bayesian inference (BI) analyses are presented above the branches as follows: ML/BI. Bootstrap values < 70% or probabilities values < 0.95 are marked with "*", and absent analysis values are marked with "-". Ex-type isolates are marked with "T". Isolates sequenced in this study are highlighted in bold and shown in color. Two isolates of *Curvicladiella cignea* (CBS 109167 and CBS 109168) were used as outgroups.

tef1

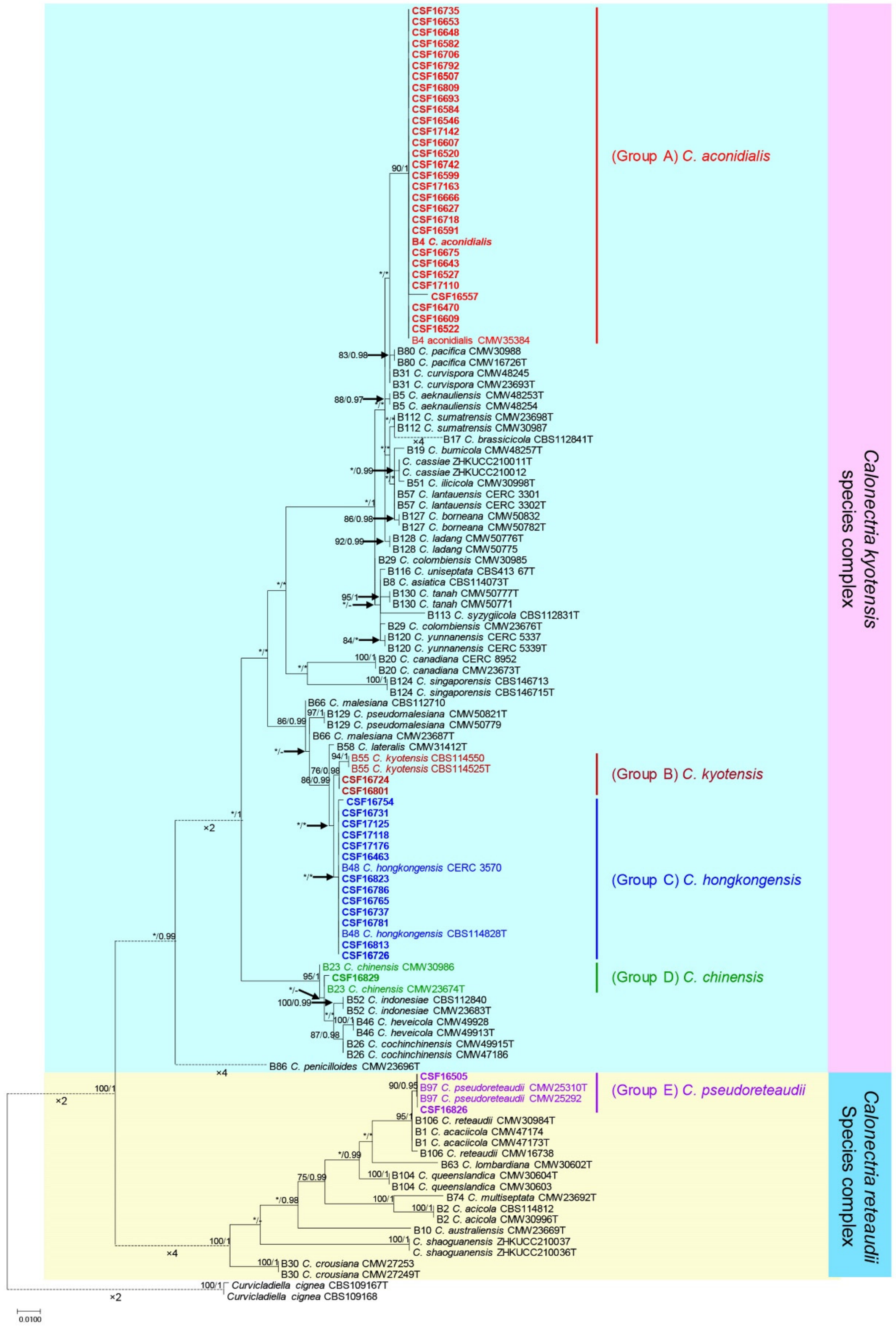

Supplementary Figure S1.

tub2

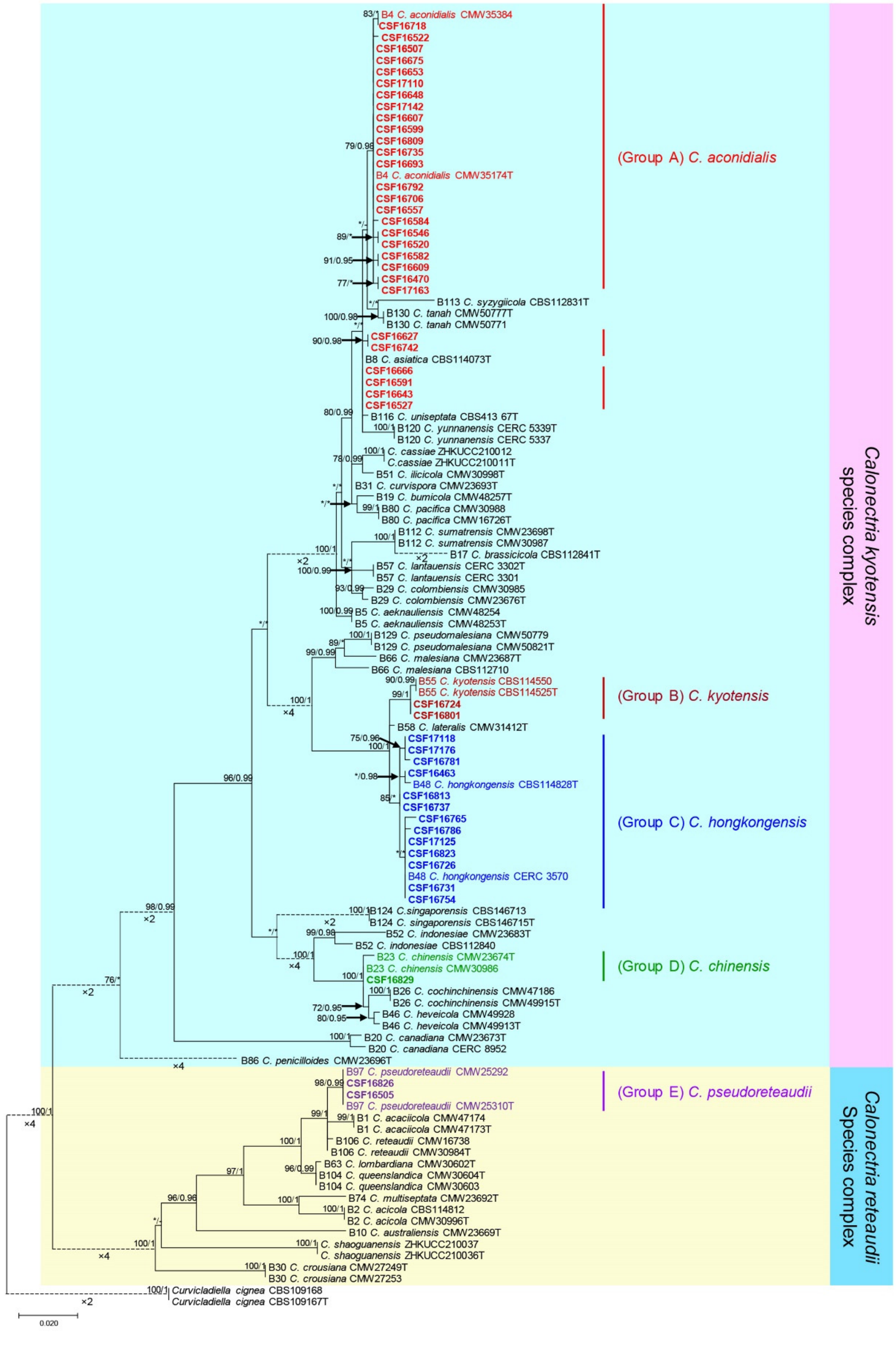

Supplementary Figure S2.

cmdA

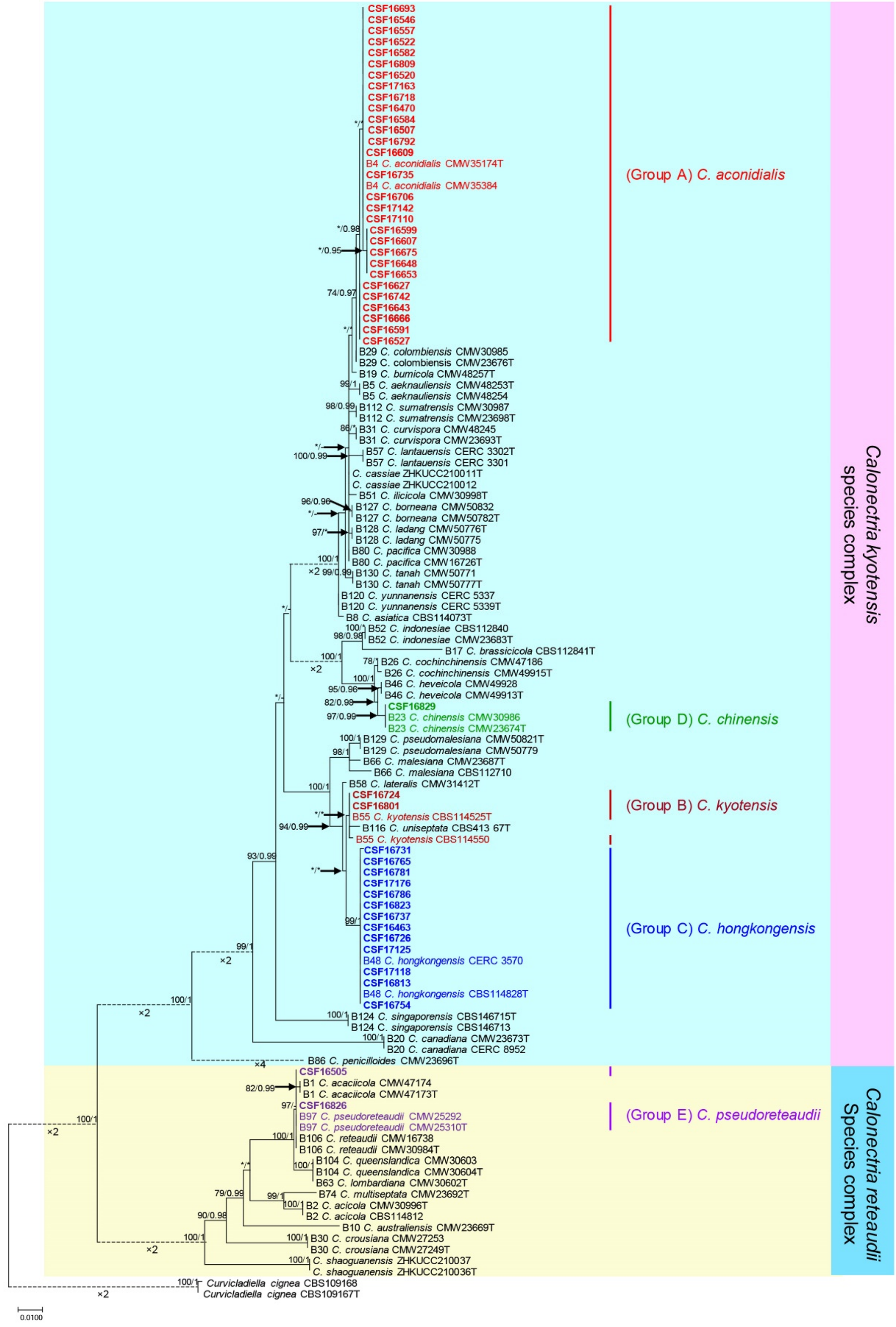

Supplementary Figure S3.

his3

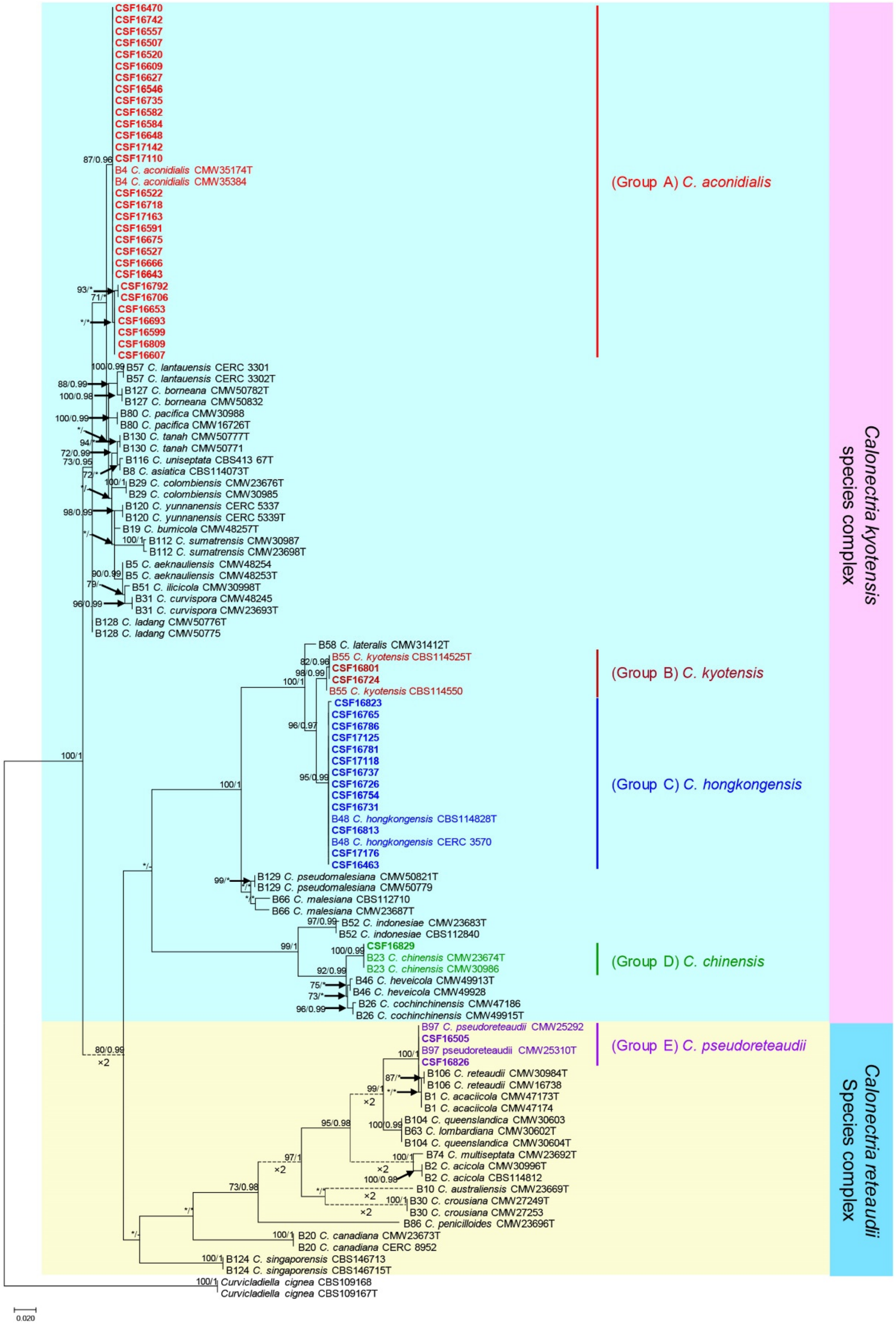

Supplementary Figure S4.
